# Spanish Rhythm Association member´s perspectives on cardiac implantable electronic device reuse in low- and middle-income countries

**DOI:** 10.1007/s10840-022-01304-y

**Published:** 2022-07-15

**Authors:** Iñigo Lorenzo Ruiz, Larraitz Gaztañaga Arantzamendi, Xabier Marichalar Mendia

**Affiliations:** 1 Nursing Department I, Faculty of Medicine and Nursing, University of the Basque Country UPV/EHU, Sarriena, Leioa, Spain; 2 BioCruces-Bizkaia Health Research Institute, Baracaldo, Spain; 3 Basurto University Hospital, Montevideo 18, 48013 Bilbao, Bizkaia Spain

**Keywords:** Pacemakers, Implantable defibrillators, Cardiac implantable electronic devices, Reuse, Opinions, Perspectives

## Abstract

**Background:**

Postmortem explanted cardiac implantable electronic devices (CIEDs) from developed countries could provide patients unable to afford new devices in low- and middle-income countries (LMIC) a treatment they lack.

This study describes the preferences of electrophysiologists and device implanting cardiologists from Spain on the management of explanted CIEDs and opinions and concerns regarding reuse in LMIC.

**Methods:**

A nationwide self-administered questionnaire was sent to members of the Spanish Rhythm Association (*n* = 1110), between December 2020 and January 2021.

**Results:**

Forty-two physician responses were obtained (response rate 5%). There was a strong preference to donate explanted devices for reuse in humans (61.9%) or animals (31%). The vast majority of the participants thought device reutilization was safe, ethical, and a reasonable alternative if a new device is not accessible. Moreover, they indicated they would be comfortable asking patients to consider post-mortem donation, and willing to implant post-mortem explanted and resterilized devices if they were unable to obtain new ones. 57.1% of respondents considered it would be beneficial for patients to have a document so they could reflect their wishes regarding device handling after their death. The most mentioned concerns regarding device reuse were malfunction (57.1%) and infection (54.8%).

**Conclusions:**

The majority of respondents support reusable CIED donation to LMIC. It would be interesting to study the feasibility of a nationwide device reutilization program.

**Supplementary Information:**

The online version contains supplementary material available at 10.1007/s10840-022-01304-y.

## Introduction

Of the 17.9 million reported deaths from cardiovascular diseases in 2019, it is estimated that approximately 75% occurred in LMIC [1]. The lack of prevention programmes and poor access to health care services, or their huge costs are some of the reasons for the high mortality from cardiovascular diseases in these countries [2].

Although CIED implantation is common in developed countries, it is not in many LMIC, partly because the cost of the devices often far exceeds the financial capacity of patients [3]. Thus, in the previous decade, the frequency of implantation in developed countries ranged from 200 to 1000, while in African countries ranged from 0.14 to 233 per million inhabitants. Due to this lack of access to CIEDs, it is estimated that approximately one million people die each year in LMIC [3].

Evidence on the reuse of CIEDs has increased in recent years and it is presented as a reasonable alternative to provide treatment when there is no possibility to access a new one [4]. Reusing CIEDs is not a new concept, as in countries such as Sweden was a common practice in the years prior to the 1990s [5]. As CIEDs must be removed from deceased patients who will be cremated, due to the risk of explosion of the devices when subjected to the high temperatures of the crematorium and approximately 21% of the explanted devices could be reusable, they could be an important resource for LMIC [6]. Furthermore, it has been stated that the reutilization of used devices is a safe practice, provided that they are reprocessed and sterilized appropriately [7]. For this reason, initiatives that recover used CIEDs in the USA and France, recondition and implant them on LMIC where national regulations allow device reutilization have emerged [8–10].

There is no device donation initiative in Spain but presumably, used devices could be donated to LMIC for reuse. Previous studies have highlighted the importance that these reusable device donation initiatives must integrate a cooperation network between manufacturers, government agencies, patients, clinicians, and funeral directors [11]. It has been reported that the vast majority of patients, potential recipients, physicians, and funeral directors support the donation of reusable devices to LMIC [12]. However, since most of these studies have been carried out in the USA, no international data on these issues are currently available.

This study aims to describe the preferences of electrophysiologists and device implanting cardiologists in Spain regarding the management of explanted devices and their opinions and concerns about the donation and reutilization in LMIC.

## Methods

This study consisted in a nationwide survey among members of the Rhythm Association, a scientific association of cardiologist physicians and other healthcare professionals, like nurses, with a special interest and involvement in rhythm and cardiac arrhythmias of the Spanish Society of Cardiology (*n* = 1110). The association’s management approved the sending of the survey through the contact email provided by the members. Invitations to participate in the online survey were sent out in three different waves separated by a period of 20 days. Data collection started at the beginning of December 2020 and ended at the end of January 2021.

The study consisted in an online self-administered form to be completed by physicians of the Rhythm Association (*n* = 832) based on similar previous studies by Hughey et al. and Logani et al. [11, 13]. The form contained 32 dichotomous, multiple choice or short answer questions. Demographic data such as gender, age, degree, professional speciality, years of experience, and type of institution of work were collected. The following questions collected data about the number of patients in follow-up, number of annual implantations of devices, number and usual handling of explanted devices, preferences about explanted devices, opinions about device advances directives, and concerns about reutilization. Finally, participants indicated their level of agreement with several positive statements on the reuse of cardiac devices by completing a Likert scale. Participants did not receive any financial compensation or benefits for completing the survey.

### Data analysis

For the description of the quantitative variables, the mean and standard deviation were used. For qualitative variables, frequencies and percentages were used. Secondly, the age was divided in two groups, according to the mean age (younger and older than the mean). Finally, the groups were compared using Pearson’s chi-square test. A *p* value < 0.05 was considered statistically significant. All analyses were performed with the Statistical Package for Social Science version 22.

## Results

We obtained 43 responses. One response identified as a nursing professional was excluded because the aim of the study was to describe the preferences of specialist practitioners. Therefore, the response rate among physicians was 5%. Table 1 shows a summary of participant’s characteristics.

**Table 1:** Participant’s general characteristics

		*n* (%)	Mean ± standard deviation
Age			49.6 ± 11.2
Sex	Male	26 (61.9)	
	Female	16 (38.1)	
Specialty	Electrophysiology	34 (81)	
	Cardiology	8 (19)	
Years of experience	1–3 years	2 (4.9)	
	4–6 years	3 (7.3)	
	> 7 years	36 (87.8)	
Performs CIED implantation and/or explantations regularly	Yes	39 (92.9)	
	No	3 (7.1)	
Average number of pacemaker implants per year			125.8 ± 114.2
Average number of defibrillator implants per year			41 ± 39.5
Average number of CIED explants per year			39.8 ± 82.6
Works in public medical center	Yes	32 (76.2)	
	No	10 (23.8)	
Works in private medical center	Yes	11 (26.2)	
	No	31 (73.8)	
Works in academic or university center	Yes	19 (45.2)	
	No	23 (54.8)	
Main work center location	Urban	38 (90.5)	
	Suburban	4 (9.5)	
Number of patients with CIEDs under follow-up	51–100	2 (4.8)	
	101–250	5 (11.9)	
	251–500	7 (16.7)	
	> 500	28 (66.7)	

*CIEDs* = cardiac implantable electronic devices

### Routine management of explanted CIEDs

The vast majority of respondents (70.7%) indicated that they had never implanted a resterilized device, while the remaining respondents indicated that they had implanted 1–10 (24.4%) or 11–25 (4.9%) resterilized devices during their career. The majority of the professionals indicated that they regularly disposed of explanted devices as health care waste (74.4%). The rest indicated that they stored them either in the consulting room or extraction site for teaching or other purposes (20.5%) or that they returned them to patients or relatives (5.1%). Table 2 summarizes the annual number of devices explanted per practitioner, ordered according to the different management options referred.

**Table 2:** Approximate mean annual number of CIEDs explanted per participant, ordered according to the different management options referred

	*n*	Mean	Standard deviation
Discarded as medical waste	38	39.74	72.559
Stored in extraction site	36	5.53	14.496
Returned to patient or family	37	2.11	5.353
Returned to manufacturer	37	2.03	4.206
Donated for animal reuse	38	0.29	1.626
Donated for human reuse	38	0.08	0.487

*CIEDs* = cardiac implantable electronic devices*. n* = number of participants that responded in each option

### Preferences regarding explanted CIEDs

Participants were asked to indicate their preference on the management of the explanted devices if such decision depended on them. Table 3 shows a summary of the responses on this question. There was a strong preference to donate explanted devices for reuse in humans (61.9%) or animals (31%). A statistically significant difference was found in the preference for storing CIEDs at the extraction site according to age group (*p* = 0.01), with the younger age group (100%) being more reluctant to store explanted devices than the older age group (73.7%). In addition, the majority (57.1%) considered that it would be beneficial for patients with implants to have an advances directives document in which they could reflect their wishes about the management of their prostheses or implants after their death.

**Table 3:** Participants’ preferences if they had a choice in the management of the explanted CIEDs

		*n* (%)	Below average age group	Above average age group	*p* value
Donation for human reuse	Yes	26 (61.9)	16 (69.6)	10 (52.6)	0.261
	No	16 (38.1)	7 (30.4)	9 (47.4)	
Donation for animal reuse	Yes	13 (31)	7 (30.4)	6 (31.6)	0.936
	No	29 (69)	16 (69.6)	13 (68.4)	
Discard as medical waste	Yes	11 (26.2)	5 (21.7)	6 (31.6)	0.472
	No	31 (73.8)	18 (78.3)	13 (68.4)	
Storage in extraction site	Yes	5 (11.9)	0 (0)	5 (26.3)	0.014
	No	37 (88.1)	23 (100)	14 (73.7)	
Return to manufacturer	Yes	4 (9.5)	3 (13)	1 (5.2)	0.613
	No	38 (90.5)	20 (87)	18 (94.8)	
Return to patient or family	Yes	3 (7.1)	3 (13)	0 (0)	0.239
	No	39 (92.9)	20 (87)	19 (100)	

*CIEDs* = cardiac implantable electronic devices

### Opinions on potential donation and reuse of CIEDs

**Fig. 1:**
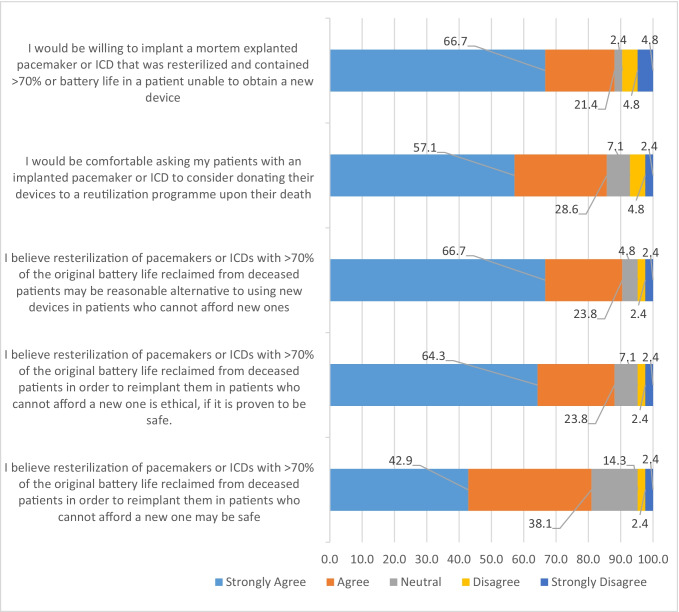
Respondents level of agreement with specific statements regarding cardiac implantable electronic device reuse. Responses are presented as percentages of respondents (*N* = 42). ICD = implantable cardioverter defibrillator

Participants indicated their level of agreement by responding on a Likert scale to various positive statements about device reuse (Fig. 1). The vast majority of the participants thought device reutilization was safe, ethical, and a reasonable alternative if a new device is not accessible. Moreover, they indicated they would be comfortable asking patients to consider post-mortem donation, and willing to implant post-mortem explanted and resterilized devices if they were unable to obtain new ones. The most commonly cited concerns about device reuse were malfunction (cited by 24 participants; 57.1%), infection (cited by 23 participants; 54.8%), ethical (cited by 4 participants; 9.5%), and legal (cited by 3 participants; 7.1%).

Finally, the vast majority of participants (85.7%) reported that they would collaborate with a CIED donation program, storing explanted devices at their respective centers for collection, analysis, cleaning, sterilization, and shipment to LMIC.

No differences were found when gender, practice type, training, location, number of patients followed, implants, or explants per year were compared with the routinary management of explanted devices, preferences regarding explanted devices or opinions on potential donation and reuse of CIEDs.

## Discussion

The most relevant contribution of this study is that the majority of Spanish Rhythm Association participants in the study support the concept of reusing CIEDs in patients who cannot afford new devices. As in the previous study by Hughey et al., there appears to be a widespread view that reimplantation of appropriately resterilized devices is a safe, reasonable and ethical alternative when a new device is not accessible [13].

Regarding the handling of explanted CIEDs, the majority of participants in our study reported either discarding the explanted devices (74.4%) or storing them in the surgery or extraction site (20.5%). These findings are partially consistent with those of the previous study by Logani et al. [11]. Devices explanted in hospitals in Spain are usually stored for didactic purposes, which may explain the differences in handling preferences of the older than average age group in our study. Moreover, the study pointed out the importance of returning explanted devices to manufacturers, so that they can be checked for possible inadvertent malfunctions [11]. However, remote monitoring is becoming increasingly common in Spain and with the integration of the National Pacemaker Registry, these defects can be detected in earlier stages [14]. Therefore, the need of returning all explanted devices to the manufacturers for malfunction detection has been minimized [11]. In addition, explanted devices are treated as biohazardous health care waste, which could explain these differences with the mentioned study [15].

Although explanted CIEDs are treated as hazardous waste, a considerable number could still have sufficient battery life and adequate function to be reconditioned and reused [16]. Although in Spain there is no legal framework on the ownership of implants, the ownership is considered to belong to the patients, so they or their relatives could claim them once they have been removed [17]. By this means, patients or relatives could recover and choose to donate their devices to a reutilization initiative, if the safe management of explanted CIEDs is ensured. Thus, on the basis of ethical and legal aspects, it has been stated the necessity to provide an advanced directive document for device donation [11]. Studies carried out in patients with CIEDs indicate that the most would be willing to sign this document [18]. In the previous study by Logani et al., only 33% of practitioners indicated that having a document for collecting these advanced directives would be beneficial, attributing the result largely to the increased time it would take for practitioners to inform and collect the advanced directives [11]. In our study, 57.1% of the participants responded that having this kind of document would be beneficial.

In our study, participants indicated their level of agreement with various positive statements on the reuse of CIEDs. As in the previous study by Hughey et al., the levels of agreement in terms of reuse and donation of devices were broadly positive [13]. Likewise, the most common concerns raised by participants surveyed about device reuse were malfunction and infection, as in the study of Hughey et al. [13]. The literature suggests that reuse of properly sterilized CIEDs in other patients is safe. In fact, the most recent meta-analysis found no significant differences in terms of infection (OR 0.98; 95% CI 0.60–1.60), malfunction (OR 1.58; 95% CI 0.56–4.48), premature battery depletion (OR 1.96; 95% CI 0.81–4.72), or device-related deaths comparing reused devices with new devices [7]. However, no randomized clinical trials have yet been conducted on this topic. So, the lack of higher quality research could be largely indicative of the data collected about these infection and malfunctioning concerns. The Project My Heart Your Heart (PMHYH), has a clinical trial underway that could shed light on these issues [19]. However, the results of this trial are not yet available. Therefore, device reutilization should only be considered in situations where a new device is not accessible, informing adequately potential recipients about the risks of reprocessed and getting the respective informed consent [20].

The vast majority of Spanish Rhythm Association participants (85.7%) stated they would support a program to donate CIEDs for reuse in LMIC, which could open the door to a national program similar to those in other countries. Taking into account the data on the advanced directives, implanting physicians could play a key role, informing patients and surrogates about potential device reuse and providing these advances directives for device donation after explant. Explanted devices could then be recovered from hospitals or funeral homes by a non-profit organization, maintaining patients’ wishes. In addition, the organization could then sterilize and recondition the devices for missions in LMIC or collaborate with PMHYH, shipping them the recovered devices, due to its expertise and larger network of professionals and hospitals involved.

Survey studies carried out in patients with devices have demonstrated a favorable view of CIED reuse [18]. It would be interesting to know the perspectives of patients with CIEDs in Spain, in order to describe if patients would be willing to donate their devices. In the most favorable scenario, a non-negligible number of devices could be recovered and provide a vital treatment for many patients unable to access a new one.

Study limitations.

The study has several limitations. First of all, the questionnaires were completed in a specific association and by email, supposing that we could not calculate how many physicians had really the chance to respond to the questionnaire. Secondly, the response rate was low (5%), so the applicability of the results at a broader level is limited. A low response rate is common in studies conducted in Heart Rhythm Association members or electrophysiologists (10–16%), but it could mean that the members who responded were more familiar and interested in CIED reuse and likely to have positive preferences on this topic [13, 21–23]. Finally, this study was carried out using quantitative methodology, allowing us to identify opinions, but not to explore the processes that lead to the generation of these opinions. It would be interesting to explore these phenomena also with a qualitative methodology.

## Conclusions

The donation of reusable CIEDs from developed countries to LMIC is an alternative that can provide vital therapy to many patients who currently lack other alternatives. A large majority of Spanish Rhythm Association respondents nationwide support the concept of donating reusable devices to patients without access to new devices. It would be interesting to describe device owners’ perspectives on potential donation, in order study the possibility of integrating a nationwide device donation initiative.

## Supplementary Information

Below is the link to the electronic supplementary material.

Supplementary file1 (DTA 47 KB)

## Data Availability

Data available on request. The data underlying this article will be shared on reasonable request to the corresponding author.
