# Supplementary material for: Spanish Rhythm Association member´s perspectives on cardiac implantable electronic device reuse in low- and middle-income countries
Source: J Interv Card Electrophysiol. 2022 Jul 15;66(5):1095–101. doi: 10.1007/s10840-022-01304-y (PMC10333412; doi:10.1007/s10840-022-01304-y)
Supplement: Supplementary file 1 — Supplementary file1 (DTA 47 KB) [file 10840_2022_1304_MOESM1_ESM.docx]

**Survey Instrument**

Questionnaire of perceptions on reuse of implantable electronic cardiac devices for Spanish members of the Rhythm Association of the Spanish Society of Cardiology.

The person completing this survey declares that:

- Has read the survey information sheet/email.

- Has been able to ask questions about the survey.

- Has received sufficient information about the survey.

- Understands that participation in the survey is voluntary.

- Understands that can withdraw from the study: Whenever, without having to give explanations and without reprisals of any kind.

- Participates freely and gives consent to the use of the data under the conditions detailed in the information sheet.

1. Date of birth:
2. Sex:
   - 1. Male
     2. Female
3. Occupation:
4. Specialty:
5. Years of experience in cardiac electrostimulation therapies:
   - 1. 1-3 years
     2. 4-6 years
     3. >7 years
6. Do you perform cardiac electrostimulation device implantation and/or explantations surgeries (such as pacemakers or ICDs) in your regular practice?
   - 1. Yes
     2. No
7. Please indicate which of the following best describes the centre where you mainly work:
   - 1. Public medical centre
     2. Private medical centre
     3. Academic and/or university centre
     4. Other (Please specify)
8. Type of location of the centre where you habitually carry out your practice:
   - 1. Urban
     2. Suburban
     3. Rural
     4. Not applicable, I do not work clinically
9. Indicate the approximate number of patients with cardiac electrostimulation devices (such as pacemakers or ICDs) under your follow-up:
   - 1. 0-50
     2. 51-100
     3. 101-250
     4. 251-500
     5. >500
     6. Not applicable, I do not work clinically
10. Indicate in numbers the approximate number of pacemakers implanted per year
11. Indicate in numbers the approximate number of defibrillators implanted per year:
12. Indicate in numbers the approximate number of defibrillators implanted per year:
13. Indicate in numbers the approximate number of total devices (pacemakers and defibrillators) explanted per year:
14. Have you ever implanted a resterilised cardiac electrostimulation device?
    - 1. Yes
      2. No
      3. Not applicable, I do not implant devices
15. If yes to the above question, how many resterilised devices have you implanted during your career?
    - 1. 1-10
      2. 11-25
      3. 26-50
      4. >50
16. What do you usually do with a cardiac electrostimulation device (such as a pacemaker), when you explant it from a patient?
    - 1. Donate it for human reuse
      2. Donate it for animal reuse
      3. Discard as medical waste
      4. Store at the extraction site
      5. Return to patient or relatives
      6. Return to manufacturer
17. Please indicate the total number of devices that you annually donate for human reuse:
18. Please indicate the total number of devices that you annually donate for animal reuse:
19. Please indicate the total number of devices that you annually discard as medical waste:
20. Please indicate the total number of devices that you annually store at extraction site:
21. Please indicate the total number of devices that you annually return to patient or relatives:
22. Please indicate the total number of devices that you annually return to manufacturer:
23. Do you think it would be beneficial to have an advances directives document for implant carriers in which they could reflect their wishes about the management of their prostheses or implants after their death?
    - 1. Yes
      2. No
24. If it depended on you, what would be your preference for the management of explanted cardiac electrostimulation devices (such as pacemakers)?
    - 1. Donate it for human reuse
      2. Donate it for animal reuse
      3. Discard as medical waste
      4. Store at the extraction site
      5. Return to patient or relatives
      6. Return to manufacturer

Please indicate your level of agreement with regard to the following statements:

1. I believe resterilization of pacemakers or ICDs with >70% of the original battery life reclaimed from deceased patients in order to reimplant them in patients who cannot afford a new one may be safe
   - 1. Strongly Agree
     2. Agree
     3. Neutral
     4. Disagree
     5. Strongly Disagree
2. I believe resterilization of pacemakers or ICDs with >70% of the original battery life reclaimed from deceased patients in order to reimplant them in patients who cannot afford a new one is ethical, if it is proven to be safe.
   - 1. Strongly Agree
     2. Agree
     3. Neutral
     4. Disagree
     5. Strongly Disagree
3. I believe resterilization of pacemakers or ICDs with >70% of the original battery life reclaimed from deceased patients may be reasonable alternative to using new devices in patients who cannot afford new ones
   - 1. Strongly Agree
     2. Agree
     3. Neutral
     4. Disagree
     5. Strongly Disagree
4. I would be willing to implant a mortem explanted pacemaker or ICD that was resterilized and contained >70% or battery life in a patient unable to obtain a new device
   - 1. Strongly Agree
     2. Agree
     3. Neutral
     4. Disagree
     5. Strongly Disagree
5. I would be comfortable asking my patients with an implanted pacemaker or ICD to consider donating their devices to a reutilization programme upon their death
   - 1. Strongly Agree
     2. Agree
     3. Neutral
     4. Disagree
     5. Strongly Disagree
6. What are your major concerns regarding post-mortem reuse of cardiac devices? (Specify all applicable)
   - 1. Infection
     2. Malfunction
     3. Religious
     4. Ethical
     5. I don’t have any
     6. Other (Specify)
7. Would collaborate with an implantable cardiac device donation programme, storing explanted devices at their respective centres for collection, analysis, cleaning, sterilization and shipment to developing countries?
   - 1. Yes
     2. No
8. If you wish to comment further, please use the following section:

Thank you for your participation.

Please don’t forget to click the submit button.
